# Percutaneous Coronary Intervention Versus Optimal Medical Therapy for Severe Ischemic Left Ventricular Systolic Dysfunction

**DOI:** 10.1016/j.mayocpiqo.2024.04.002

**Published:** 2024-05-19

**Authors:** Ruth A. Mathew Kalathil, Akshay Machanahalli Balakrishna, Ahmed El-Shaer, Andrew M. Goldsweig, Khagendra Dahal, Saraschandra Vallabhajosyula, Ahmed Aboeata

**Affiliations:** a Department of Medicine, Creighton University School of Medicine, Omaha, NE; b Division of Cardiovascular Medicine, Department of Medicine, Creighton University School of Medicine, Omaha, NE; c Department of Cardiovascular Medicine, Baystate Medical Center, Springfield, MA; d Division of Cardiology, Department of Medicine, Warren Alpert Medical School of Brown University and Lifespan Cardiovascular Institute, Providence, RI

## Abstract

Coronary artery disease is the most common cause of heart failure, which is the leading cause of cardiovascular-related death worldwide. There are insufficient data to make strong recommendations for percutaneous coronary intervention (PCI) in patients with severe ischemic left ventricular systolic dysfunction (LVSD). In that context, we performed a meta-analysis to compare the outcomes of PCI with those of optimal medical therapy alone in patients with severe ischemic LVSD. A systematic search was conducted in PubMed, EMBASE, and ClinicalTrials.gov from inception to December 2023. Our outcome of interest was all-cause mortality in patients undergoing PCI vs medical therapy. We used random effects models to aggregate data and to calculate pooled incidence and relative risk with 95% CIs. Four studies including 2 randomized controlled trials with 2080 patients (PCI, 1082; optimal medical therapy, 998) were included. All-cause mortality did not differ significantly between the groups: 168 patients (15.5%) in the PCI group vs 200 patients (20.0%) in the optimal medical therapy group (relative risk, 0.88; 95% CI, 0.75-1.09; *P*=.25). In conclusion, the available evidence indicates that PCI does not improve all-cause mortality in patients with severe LVSD without lifestyle-limiting anginal symptoms. Further data are needed to identify subgroups of patients better served by each modality.

Heart failure is the leading cause of cardiovascular-related death worldwide, and coronary artery disease (CAD) is the principal cause of heart failure.^1^^,^^2^ The prospect of reviving viable but ischemic or hibernating myocardium through revascularization has fueled substantial research.^1^^,^^3^ Current multisociety guidelines favor revascularization with coronary artery bypass grafting (CABG) over optimal medical therapy (OMT) alone to improve all-cause mortality in patients with severe ischemic left ventricular systolic dysfunction (LVSD) with ejection fraction of less than or equal to 35%.^3^ Despite the guidelines, patients with severe ischemic LVSD frequently do not undergo CABG owing to operative risks, patient reluctance, or suboptimal anatomy for CABG.^3^ Percutaneous coronary intervention (PCI) offers an attractive alternative modality to achieve revascularization in patients with severe LVSD.^1^^,^^3^ Current American and European guidelines have insufficient data to make strong recommendations for PCI in patients with severe LVSD. In that context, we performed a meta-analysis to compare the outcomes of PCI with those of OMT in patients with severe ischemic LVSD.

A systematic review was conducted in PubMed, EMBASE, and ClinicalTrials.gov from inception through December 2023. The study protocol was submitted to the international prospective register of systematic reviews for registration and did not require institutional review board approval because publicly available data were used. Two reviewers (R.A.M.K., A.M.B.) independently extracted pertinent data into data collection tables. All discrepancies between the reviewers were resolved by consultation with other authors. Severe ischemic LVSD was defined as significantly impaired left ventricular dysfunction (ejection fraction ≤35%), resulting from CAD. The primary outcome of interest was all-cause mortality. We used the Mantel-Haenszel random-effect model to estimate risk ratios and 95% CIs. The quality and risk of bias of included studies were evaluated using the Newcastle-Ottawa scale quality score and the Cochrane risk of bias tool, respectively. All analyses were performed using the Cochrane Review Manager (RevMan) version 5.4.

Four studies^1^^,^^3^, ^4^, ^5^ including 2 randomized controlled trials (RCTs) with 2080 patients (PCI, 1082; OMT, 998) were included. The overall risk of bias among the included trials was low (*I*^*2*^=12%). All-cause mortality did not differ significantly between the groups: 168 patients (15.5%) in the PCI group vs 200 patients (20.0%) in the OMT group (risk ratio, 0.88; 95% CI, 0.75-1.09; *P*=.25) (Figure). The follow-up duration in the studies varied from 2 to 3 years for all studies except LaBarbera et al^5^ where the reported outcome was in-hospital mortality. Sensitivity analysis performed using the leave-1-out method yielded similar results.

**Figure:**
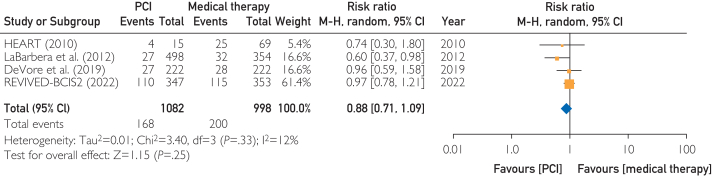
Forest plot of all-cause mortality comparing PCI with OMT in ischemic cardiomyopathy with severe LVSD. LVSD, left ventricular systolic dysfunction; HEART, The Heart Failure Revascularization Trial; M-H, Mantel-Haenszel; OMT, optimal medical therapy; PCI, percutaneous coronary intervention, REVIVED-BCIS2, Revascularization for Ischemic Ventricular Dysfunction-British Cardiovascular Intervention Society-2.

In this meta-analysis of 4 studies, we found that, among patients with severe ischemic LVSD, there was no significant difference in all-cause mortality between revascularization with PCI and OMT alone (*P*=.25). Both RCTs included in this meta-analysis identified patients with a substantial volume of viable myocardium before undergoing revascularization; however, this characteristic did not translate to improved survival in this patient population. However, as previously noted, patients in the REVIVED-BCIS-2 (Revascularization for Ischemic Ventricular Dysfunction-British Cardiovascular Intervention Society-2) trial had a low anginal burden.^1^ Therefore, the results may not apply to patients with ischemic LVSD and lifestyle-limiting angina. Our results contrast with the findings of the Surgical Treatment for Ischemic Heart Failure trial, which reported an association between surgical revascularization via CABG and improved survival among patients with LVSD and extensive CAD at 10-year follow-up.^6^ Possible explanations for the lack of benefit with PCI include considerable improvements in OMT (angiotensin receptor + neprilysin and SGLT2 inhibitors and increased use of biventricular pacemakers/defibrillators), shorter duration of follow-up, lower-than-expected ischemic and anginal burden, and less extensive CAD. Even the landmark Surgical Treatment for Ischemic Heart Failure trial found no survival benefits with surgical revascularization at 5-year follow-up and reported a positive survival outcome only after 10 years.^6^ Other potential factors that can influence outcomes in PCI include the timing of intervention. Ischemic evaluations are underused in patients with LVSD, delaying the timing of intervention. Limitations in technology around the time these studies were performed could also play a role. In our meta-analysis, we included 2 RCTs, the Heart Failure Revascularisation trial and the REVIVED-BCIS2 trial, which included patients with some form of viability testing demonstrating more than 4 segments of viable myocardium in a 17-segment model. Despite this, there was no improvement in outcomes with PCI compared with those using medical management, which aligns with the findings from those of previous studies.

This meta-analysis is limited by the small number of RCTs, intrinsic limitations of the individual studies (including short follow-up and low event rates), and the lack of patient-level data, which prevented us from identifying specific subgroups that may benefit from revascularization by PCI. Of the 4 studies included in this trial, only 1 (REVIVED-BCIS2 trial) has reported a change in left ventricular ejection fraction. There was no change in left ventricular function noted between the PCI cohort and the OMT arm at 6-month or 12-month follow-ups. In aggregate, the available evidence indicates that PCI does not improve all-cause mortality in patients with severe LVSD without lifestyle-limiting anginal symptoms. Further data are needed to identify subgroups of patients better served by each modality.

## Potential Competing Interests

Dr Goldsweig reports consulting for Philips and Inari Medical and speaking for Philips and Edwards Lifesciences.
